# Etiology of Persistent Microalbuminuria in Nigeria (P_MICRO study): protocol and study design

**DOI:** 10.1186/s12879-022-07531-y

**Published:** 2022-07-04

**Authors:** C. William Wester, Bryan E. Shepherd, Usman J. Wudil, Baba Maiyaki Musa, Donna J. Ingles, Heather L. Prigmore, Faisal S. Dankishiya, Aima A. Ahonkhai, Bukar A. Grema, Philip J. Budge, Ayumi Takakura, Opeyemi A. Olabisi, Cheryl A. Winkler, Jeffrey B. Kopp, Joseph V. Bonventre, Christina M. Wyatt, Muktar H. Aliyu

**Affiliations:** 1 Vanderbilt Institute for Global Health (VIGH), 2525 West End Avenue, Suite 750, Nashville, TN 37203-1738 USA; 2 Department of Medicine, Division of Infectious Diseases, Vanderbilt University Medical Center (VUMC), Nashville, TN 37203-1738 USA; 3 Department of Biostatistics, Vanderbilt University Medical Center (VUMC), Nashville, TN USA; 4 Department of Medicine, Aminu Kano Teaching Hospital (AKTH), Kano, Nigeria; 5 Africa Center of Excellence for Population Health and Policy, Bayero University, Kano, Nigeria; 6 Department of Family Medicine, Aminu Kano Teaching Hospital (AKTH), Kano, Nigeria; 7 Department of Medicine, Infectious Diseases Division, Washington University in St. Louis School of Medicine, St. Louis, MO USA; 8 Brigham and Women’s Hospital, Division of Renal Medicine, Boston, MA USA; 9 Department of Medicine, Harvard Medical School, Boston, MA USA; 10 Department of Medicine, Division of Nephrology, Duke University School of Medicine, Duke Clinical Research Institute, Durham, NC USA; 11 Basic Research Laboratory, Frederick National Laboratory for Cancer Research, Frederick, MD USA; 12 Kidney Diseases Branch, National Institute of Diabetes and Digestive and Kidney Diseases (NIDDK), Bethesda, MD USA; 13 Department of Health Policy, Vanderbilt University Medical Center (VUMC), Nashville, TN USA

**Keywords:** *APOL1*, Microalbuminuria, Kidney disease, HIV, Nigeria

## Abstract

**Background:**

Microalbuminuria is an independent risk factor for cardiovascular and kidney disease and a predictor of end organ damage, both in the general population and in persons with HIV (PWH). Microalbuminuria is also an important risk factor for mortality in PWH treated with antiretroviral therapy (ART). In the ongoing Renal Risk Reduction (R3) study in Nigeria, we identified a high prevalence of microalbuminuria confirmed by two measurements 4–8 weeks apart in ART-experienced, virologically suppressed PWH. Although Stage 1 or 2 hypertension and exposure to potentially nephrotoxic antiretroviral medications were common in R3 participants, other traditional risk factors for albuminuria and kidney disease, including diabetes, *APOL1* high-risk genotype, and smoking were rare. Co-infection with endemic pathogens may also be significant contributors to albuminuria, but co-infections were not evaluated in the R3 study population.

**Methods:**

In Aim 1, we will cross-sectionally compare the prevalence of albuminuria and established kidney disease risk factors in a cohort of PWH to age- and sex-matched HIV-negative adults presenting for routine care at the Aminu Kano Teaching Hospital in Kano, Nigeria. We will leverage stored specimens from 2500 R3 participants and enroll an additional 500 PLWH recently initiated on ART (≤ 24 months) and 750 age- and sex-matched HIV-negative adults to determine the contribution of HIV, hypertension, and other comorbid medical conditions to prevalent albuminuria. In Aim 2, we will follow a cohort of 1000 HIV-positive, ART-treated and 500 HIV-negative normoalbuminuric adults for 30 months to evaluate the incidence and predictors of albuminuria.

**Discussion:**

The findings from this study will support the development of interventions to prevent or address microalbuminuria in PWH to reduce kidney and cardiovascular morbidity and mortality. Such interventions might include more intensive monitoring and treatment of traditional risk factors, the provision of renin-angiotensin aldosterone system or sodium-glucose cotransporter-2 inhibitors, consideration of changes in ART regimen, and screening and treatment for relevant co-infections.

**Supplementary Information:**

The online version contains supplementary material available at 10.1186/s12879-022-07531-y.

## Background

In some forms of kidney disease, such as diabetic nephropathy, the earliest detectable abnormality is often microalbuminuria. Albuminuria is an independent risk factor for cardiovascular complications and is a predictor of end organ damage including chronic kidney disease (CKD) [1–4]. Even very low levels of albuminuria, assessed as urine albumin/creatinine ratio (uACR), of 10–30 mg/g) have been associated with adverse cardiovascular and kidney events. [5, 6] Microalbuminuria is used in the early detection of disease states characterized by microvascular dysfunction, such as preeclampsia and diabetic nephropathy, as well as medication-induced nephrotoxicity [7, 8]. Micro- and macroalbuminuria are also important risk predictors for mortality in antiretroviral therapy (ART)-treated PWH, likely as a marker for inflammation and endothelial activation [9, 10]. Albuminuria has been associated with decreased CD4 cell counts and higher plasma viral loads in PWH, as well as increased systemic T-cell activation and more rapid progression to AIDS [5, 11, 12].

In recently published data from our NIDDK-funded R3 study [13, 14] among PWH, ages 18–69 years who were on ART for ≥ 6 months, ~ 40% of 2500 enrolled participants had some level of persistent albuminuria. Why is albuminuria so common in this HIV-positive, ART-experienced population? We hypothesized that genetics play an important role, given the high prevalence of *APOL1* renal risk variants in West Africa [13, 14]. However, genotyping in the R3 study revealed lower than anticipated prevalence of the high-risk (HR) *APOL1* genotype (6.2%), which has been associated with kidney disease risk [13]. The high rates of albuminuria observed in the R3 study population likely reflect a mix of both reversible microvascular endothelial injury (inflammation driven) and non-reversible podocyte damage (irreversible and progressive to CKD). The current study is designed to evaluate additional potential contributors to the high observed rates of albuminuria observed in PWH in Nigeria. *APOL1* high risk genotype and traditional risk factors (e.g., hypertension, diabetes, smoking) do not fully explain the high rates of albuminuria in the R3 study population, although hypertension is likely to be a significant contributing factor. In a prior study in Nigeria, adults with high normal blood pressure (BP) (systolic blood pressure [SBP] 130–139 mmHg and/or diastolic blood pressure [DBP] 85–89 mmHg) had higher rates of microalbuminuria (12.9% vs. 4.1%) and cardiovascular disease risk factors than those with optimal BP. Sickle cell disease and sickle cell trait are also common, with sickle cell trait affecting 1 in 12 African Americans and nearly 300 million people worldwide [15–17]. Although sickle cell trait is often considered a benign condition, kidney manifestations may include impaired urinary concentrating ability, hematuria, and papillary necrosis [17–21]. Although the relationship of sickle cell trait to long-term CKD has not been firmly established, prior studies have showed a higher-than-expected prevalence of sickle cell trait among participants with end stage kidney disease (ESKD). [15, 22, 23]

Other factors that may contribute to albuminuria can be broken down into three main categories: i) *direct kidney injury* (i.e., exposure to the ARV medication TDF, which can cause tubulopathy); ii) *immune activation/inflammation* from HIV or specific viral pathogens (e.g.*, Cytomegalovirus* [CMV], hepatitis B or C) and/or other endemic co-infections (e.g., parasitic infestations, tuberculosis); and iii) *environmental factors/exposures* (*i.e*., traditional medications, heavy metals, etc.). This study will include an in-depth evaluation of direct kidney injury and immune activation/inflammation. Depending on results, future studies may evaluate potential environmental exposures.

Because R3 participants were ART-experienced, with the majority receiving TDF at some point in their course, another important research question is to determine whether albuminuria is *tubular* or *glomerular* in etiology. Tenofovir is an effective ARV medication, widely used in first-line ART regimens [24, 25]. Although data suggest that it is not universally associated with kidney toxicity [26–30], it can be associated with renal tubular dysfunction, including Fanconi syndrome [25, 30–33]. This is especially important in our study setting, where the alternative formulation of tenofovir, tenofovir alafenamide (TAF), which has a more favorable bone and kidney toxicity profile, is not routinely available.

Despite improved patient survival with ART [34, 35], non-AIDS defining conditions such as cardiovascular and kidney disease and non-AIDS-defining malignancies are more prevalent among ART-treated HIV-positive than HIV-negative adults [34, 36–39]. While factors such as smoking and ARV-related toxicity explain some of the increased risk for these events [34, 40], excess immune activation, inflammation, and poor immune restoration [34, 41–51] have also been shown to predict disease. Chronic immune activation and inflammation are hallmarks of HIV infection. [34, 51, 52]

A diverse array of plasma biomarkers [53], including indices of T-cell activation [34, 54, 55], soluble tumor necrosis factor receptor I (sTNFR-I), sTNFR-II, and interleukin-6 (IL-6) [52, 56] are elevated in untreated HIV infection and often remain elevated among ART-treated adults who are fully virologically suppressed [34, 37, 47, 57, 58]. Many mechanisms have been proposed to explain this inflammatory state, including the direct effects of HIV replication (specifically viral protein production), HIV-mediated destruction of mucosal barriers with chronic exposure to gut microbial elements, and coinfections (e.g., CMV) [34, 59, 60]. Recent studies have shown that morbidity and/or mortality are associated with serum inflammation biomarkers, including IL-6, fibrinogen, C-reactive protein, soluble CD14 (sCD14), D-dimer, and cystatin C [34, 41, 42, 45, 46, 48, 49, 61]. Tenorio et al. showed that higher levels of IL-6, sTNFR-I, sTNFR-II, D-dimer, and sCD14 at baseline and pre-event time points were associated with non-AIDS-related morbidity or death [34]. These effects were independent of traditional risk factors, other comorbid conditions, age, ART regimen, and ART-associated immunologic recovery [34].

Systemic inflammation in persons with HIV may also occur in the setting of co-infections. For example, inflammation from parasitic infections, including malaria may cause clinical manifestations [62–69]. Parasitic infections can present with a range of glomerular diseases, most commonly membranoproliferative glomerulonephritis, while membranous glomerulopathy, focal segmental glomerulosclerosis (FSGS), and minimal-change disease are also occasionally seen [67, 68]. In West Africa, chronic parasitic infestations such as schistosomiasis (*Schistosoma haematobium and mansoni*), filariasis (*Wuchereria bancrofti*), onchocerciasis (*Onchocerca volvulus*), strongyloidiasis (*Strongyloides stercoralis*), and Loiasis (*Loa loa*) are potential infectious causes of nephrotic and nephritic syndromes [67, 68, 70]. In addition to overt kidney injury, parasitic infections may also promote local or systemic inflammation, as indicated by elevated levels of inflammatory biomarkers [69, 70].

Co-infection with certain viruses, such as CMV and hepatitis B and C, are common among PWH [71]. Among PWH, CMV seropositivity has been associated with persistent CD8 T-cell elevation and increased risk of developing non-AIDS comorbidities, despite long-term ART [72, 73]. CMV-seropositive individuals with and without HIV have increased epithelial gut damage, microbial translocation, and inflammation. However, little is known about the prevalence and impact of CMV in Nigeria. The estimated prevalence of hepatitis B virus (HBV) and hepatitis C virus (HCV) is 7.6% and 1.7%, respectively, among Nigerian adults (15–64 years of age) [74]. Various forms of kidney injury have been described in relation to HBV, including membranous nephropathy, membranoproliferative glomerulonephritis, and polyarteritis nodosa [69, 75]. In addition to these viral co-infections, mycobacterial infections may present with a diverse spectrum of kidney injuries, including post-infectious glomerulonephritis. Co-infection with *M. tuberculosis* (TB)*,* which is endemic in West Africa, particularly among HIV-positive individuals, is also associated with multiple nephropathies including chronic interstitial nephritis and proliferative glomerulonephritis [76].

To better understand the high rates of microalbuminuria in our population, we plan to test the following overarching hypothesis: hypertension (including pre-hypertension), immune activation from co-infections, and cumulative, long-term exposure to potentially nephrotoxic ARV medications contribute to the high rates of persistent microalbuminuria in these ART-experienced adults. To evaluate this overarching hypothesis, we plan the following specific aims, with each aim having its own additional supporting hypothesis or hypotheses:

1. To compare the prevalence of albuminuria and established kidney disease risk factors in a large cohort of PWH to age- and sex-matched HIV-negative adults presenting for routine medical care at the Aminu Kano Teaching Hospital (AKTH) in Kano, Nigeria. ***Hypothesis***: *HIV-negative adults will have lower prevalence of albuminuria, as well as lower prevalence of hypertension and hepatitis B and C co-infection, when compared to PWH.*
2. To determine the role that hypertension and other comorbid medical conditions (e.g., sickle cell trait or disease, immune activation/inflammation from parasitic, viral, and bacterial infections, and exposure to potentially nephrotoxic ARV medications), have on the risk for development of albuminuria. ***Hypotheses***: *a) Participants with hypertension (controlling for age and stage of hypertension) will have significantly higher rates of albuminuria; b) ongoing inflammation, as evidenced by higher levels of traditional and kidney-specific inflammatory biomarkers, will be associated with higher rates of albuminuria; and c) cumulative exposure to TDF significantly contributes to risk of incident albuminuria among PWH.*

## Methods/design

### Aim

The goal of this study is to better understand the factors contributing to the high rates of microalbuminuria observed in virologically suppressed PWH in Nigeria. By establishing an age- and sex-matched HIV-negative cohort and screening both study populations for cofactors including viral (CMV, hepatitis B & C), parasitic, and mycobacterial (TB) coinfections that may lead to immune activation/inflammation, as well as long-term exposure to potentially nephrotoxic ARV medications (particularly TDF) among PWH, we anticipate our study findings to inform the development of specific interventions to address albuminuria and result in reductions of kidney and cardiovascular morbidity and mortality.

### Setting

**Fig. 1:**
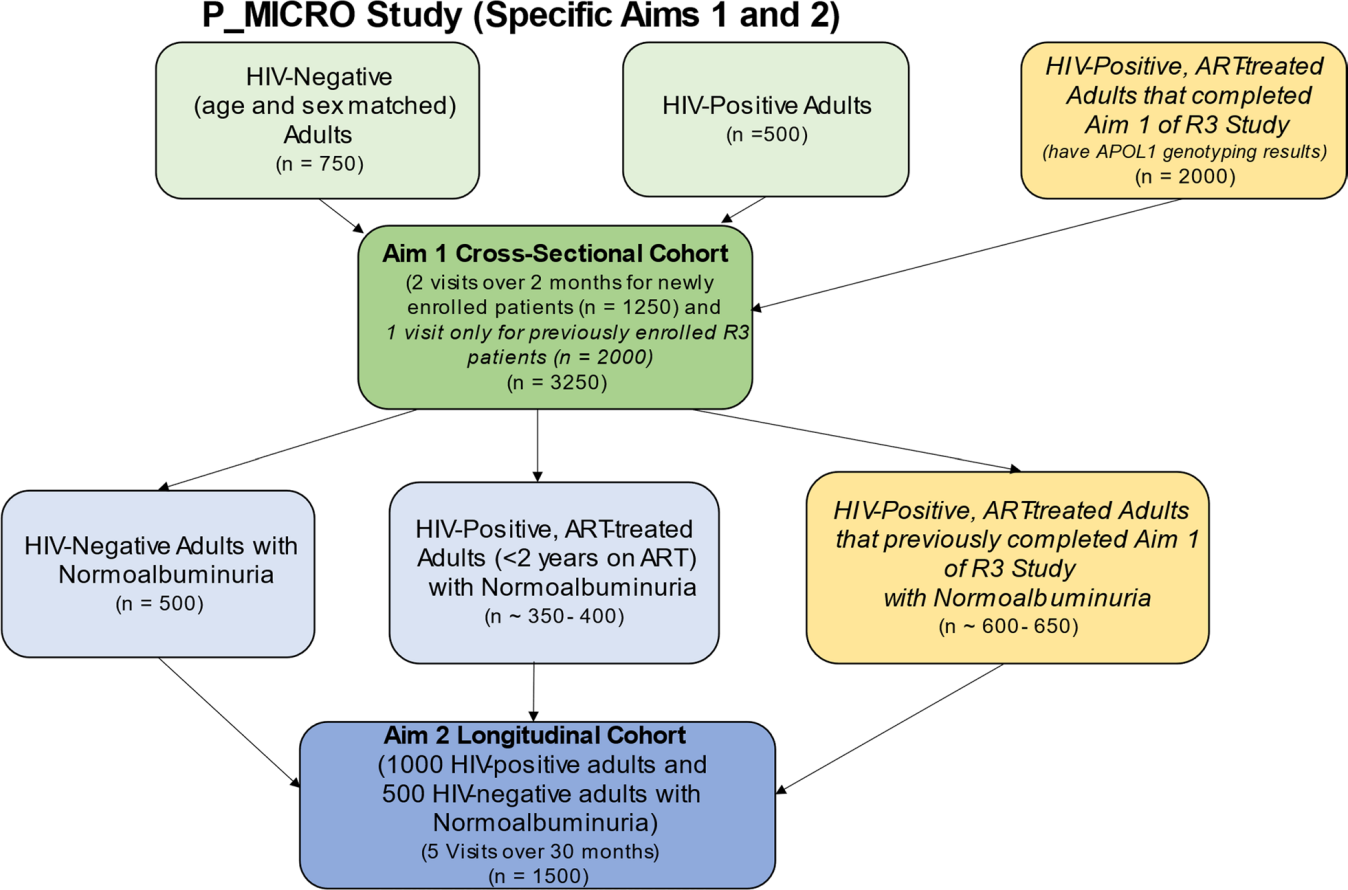
Enrollment Chart

This study will be conducted in the U.S. President’s Emergency Plan for AIDS Relief (PEPFAR)-funded HIV clinic at AKTH in Kano, a state in northwest Nigeria. Kano is the most populous state in Nigeria and has an HIV prevalence of 1.3% [77]. AKTH is a large tertiary center that provides care for more than 10,000 adult PWH. Potentially eligible study participants for this study will be identified from 2 main clinics located on the grounds of AKTH; (i) the HIV-negative adults will be identified from the General Outpatient Department (OPD) and the (ii) newly enrolled PWH plus the R3 participants called back (Fig. 1) will be identified from the Prof. S.S. Wali Virology Centre. AKTH has longstanding collaborations with Vanderbilt University Medical Center and is the site for multiple clinical trials, primarily funded by the U.S. National Institutes of Health (NIH) and the Bill and Melinda Gates Foundation.

### Study design

The study includes 2 specific aims; a cross-sectional Aim (Aim 1) and a longitudinal Aim (Aim 2) (see below and Fig. 1).

### Specific aim 1

#### Study population

The *inclusion criteria* are as follows:

- 18–69 years of age
- Willing and able to provide informed consent.

The *exclusion criteria* are:

- Pregnant (all women with reproductive potential will undergo urine pregnancy testing)
- Known congenital kidney disease or autoimmune disease (e.g., systemic lupus erythematosus) that can cause albuminuria

#### Study procedures

##### HIV-negative adults

Trained study staff will present the recruitment script at the time of screening. After subjects provide informed consent, HIV-negative participants will be tested for HIV in accordance with existing national standard/policy as follows:

- Participant will undergo point-of-care (POC) HIV rapid testing (via finger prick)
- If their POC HIV rapid test is **positive**, the Determine HIV-1/2 assay kit (Abbott Laboratories) with UniGold test (Trinity Biotech) will be used as the confirmatory test. If needed, the HIV-1/2 STAT-PAK Dipstick assay (Chembio Diagnostic Systems will be used as tiebreaker.
- If their confirmatory HIV testing is **positive**, they are not eligible to continue in the study but will be informed of their status and will receive appropriate counseling by study staff, all of whom have IRB training and extensive HIV counseling experience. They will be referred for care per existing national standards. If the HIV test is **negative**, participants will be asked to complete a brief risk assessment, asking, “*Have you engaged in any high risk (i.e., unprotected intercourse with a person with known HIV or unknown HIV status, *etc*.) one or more times within the prior 4–6 weeks*?” (**NOTE**: those who are currently healthcare workers will also be asked if they have had any needlestick and/or mucous membrane exposures from someone with known HIV-positive or unknown HIV status within the prior 4–6 weeks). Based on responses to the risk assessment question, subjects deemed *high-risk* (answering **yes** to any question), will undergo viral load (VL) testing.
- ALL consenting subjects with a **negative** POC HIV rapid test at their screening visit, regardless of their responses to the above risk assessment question, will be asked to provide urine specimens at the study clinic (visits 1 and 2) for uACR measurement. They will also have a blood specimen obtained for genetic testing (at visit 2; see Table 1).
- When VL results are available (in ~ 2–3 weeks), participants will be called back to receive results and counseling to reduce transmission risk or for referral to care if HIV-positive. Screened high-risk individuals having a detectable VL (> 50 copies/mL) are not eligible for the study and will be provided counseling and referral to care, as detailed above.

**Table 1:** Procedures for cross-sectional cohort of age- and sex-matched HIV-negative participants (n = 750), plus the newly enrolled HIV-positive, ART-treated adults (on ART for < 24 months) (n = 500)

Visit 1	Visit 2 (4–8 weeks after Visit 1)
– Screening, informed consent – HIV testing (HIV-negative participants only) – Blood pressure measurement – Diabetes screening^a^ – Enrollment (if meet eligibility criteria) – Participant to provide urine specimen at study clinic on day of visit (for 1st uACR and for urine albumin-to-protein ratio (uAPR) (if HIV-positive))	– Participant to provide urine specimen at study clinic on day of visit (for 2nd uACR) – Specimen collection (~ 6 tubes; ~ 30 mL total) i) *Genotyping* (Two DBS cards) ii) *Serum creatinine* (3 mL gold top tube) iii) *HBV*^*b*^* and HCV testing*^c^ (Two 3.5 mL gold top tubes) iv) *CMV testing*^d^ (One 3.5 mL gold top tube) v) *Additional specimens to store* (Two urine aliquots: Two 3 mL EDTA tubes; toenail/hair samples from consenting patients)

^a^Screen for diabetes (if symptomatic, e.g., polyuria, polydipsia, weight loss), perform fasting/random glucose test (point-of-care device) and if ≥ 6.1 mmol/L (110 mg/dL), then patient has possible diabetes, and is referred to medical provider/clinic for further evaluation

^b^Screen initially with hepatitis B surface antigen, and if positive, perform hepatitis B e Ag and hepatitis B DNA (viral load)

^c^Screen initially with hepatitis C antibody (due to anticipated low prevalence (< 1%), we will test a random sample of 200 HIV-positive adults and perform additional screening (stored samples) only if prevalence > 2%)

^d^Screen for Cytomegalovirus (CMV) using IgG antibody tests

Visit specifics are outlined in Table 1 (see above) for the newly enrolled 1250 study participants (500 HIV-positive adults on ART < 24 months plus the age- and sex-matched 750 HIV-negative adults) and in Table 2 (see below) for the 2,000 R3 participants that will be called back. Cross-sectional study visits will be completed by 1250 total participants, including 500 HIV-positive, ART-treated adults (18–69 years of age) on ART for < 24 months, plus 750 age- and sex-matched HIV-negative adults (Fig. 1). We will measure urinary albumin and creatinine levels to calculate a mean (two-sample) uACR. In the 500 HIV-positive adults, we will also measure urine protein (once at Visit 1) to perform a urine albumin-to-protein ratio (uAPR). In addition, we will call back R3 participants who completed screening but were not enrolled in the randomized trial for additional measurements. We anticipate obtaining these additional measurements from ~ 2000 R3 participants (conservatively assuming 90% re-consent). In participants called back from R3, we will obtain one uACR measurement (to confirm their current albuminuria status), one uAPR measurement (to evaluate the potential impact of current/prior TDF exposure), and serum creatinine. We will leverage archived specimens for HBV and CMV serology testing. HCV antibody testing will be performed if we identify a higher-than-expected prevalence in a random sample of 200 PWH.

**Table 2:** Procedures for one time cross-sectional study visit for the HIV-positive, ART-treated adults that participated in the Renal Risk Reduction (R3) study and now called back (n = 2,000)

Visit 1	
-Screening, informed consent -Blood pressure measurement -Diabetes screening^a^ -Enrollment (if meet eligibility criteria) -Participant to provide urine specimen at study clinic on day of visit (for uACR and uAPR)	-Participant to provide urine specimen at study clinic on day of visit (for one-time uACR and uAPR) **Specimen collection** (~ 3 tubes; ~ 15 mLs total) i) *Serum creatinine* (3 mL gold top tube) ii) *HBV*^*b*^* and HCV testing*^c^ (leverage existing stored plasma aliquots) iii) *CMV testing*^d^ (leverage existing stored plasma aliquots) iv) *Additional specimens to store* (Two urine aliquots: Two 3 mL EDTA tubes; toenail/hair samples from consenting patients)

^a^Screen for diabetes (if symptomatic, e.g., polyuria, polydipsia, weight loss), perform fasting/random glucose test (point-of-

care device) and if ≥ 6.1 mmol/L (110 mg/dL), then patient has possible diabetes, and is referred to medical

provider/clinic for further evaluation

^b^Screen initially with hepatitis B surface antigen, and if positive, perform hepatitis B e Ag and hepatitis B DNA (viral load)

^c^Screen initially with hepatitis C antibody (due to anticipated low prevalence (< 1%), we will test a random sample of 200

HIV-positive adults and perform additional screening (stored samples) only if prevalence > 2%)

^d^Screen for Cytomegalovirus (CMV) using IgG antibody tests

##### Data collection and privacy considerations

Following enrollment, study participants will be assigned unique study identification numbers for deidentified collection of study data elements. Study visits with trained personnel will be held in private interview rooms located at AKTH. We will use REDCap for standardized, HIPAA-compliant, secure data collection using case report forms (CRFs).

#### Study measures

##### Urine albumin-to-creatinine ratio (uACR)

*In* the Aim 1 cross-sectional arm, we will determine an average uACR (based on 2 uACR measurement values obtained 4–8 weeks apart) for the 500 newly enrolled HIV-positive, ART-treated adults (on ART < 24 months) plus the 750 age- and sex-matched HIV-negative controls. We will obtain one confirmatory uACR from the 2000 HIV-positive, ART-treated adults that we will call back from the R3 Study; all these participants have two uACR measurements from the R3 screening phase.

##### Urine albumin-to-protein ratio (uAPR)

As a rough estimate of glomerular vs. tubular injury). In the Aim 1 cross-sectional arm, we will obtain one uAPR from all HIV-positive ART-treated adults (including the 500 newly enrolled HIV-positive adults [on ART < 24 months] plus the 2000 ART-experienced PWH who we call back from the R3 Study.

##### Kidney function

(Estimated glomerular filtration rate (eGFR) measurement (serum) (i.e., creatinine); In the Aim 1 cross-sectional arm, we will obtain one serum creatinine (SCr) measurement from each participant.

##### Hepatitis B and C infection status

Hepatitis B infection status to be ascertained via serum hepatitis B surface antigen testing, with hepatitis C infection status to be ascertained via serum hepatitis C antibody testing.

##### Cytomegalovirus (CMV)

Infection status. Ascertained via serum CMV IgG antibody (Ab) testing.

##### Hypertension

Study participant’s blood pressure will be classified using current Joint National Commission (JNC) 8 guidelines; namely: *Normal*: Less than 120/80 mm Hg; *Elevated*: Systolic between 120–129 and diastolic less than 80; *Stage 1*: Systolic between 130–139 or diastolic between 80–89; and *Stage 2*: Systolic at least 140 or diastolic at least 90 mm Hg.

#### Study outcomes

1. Prevalence and extent of albuminuria (uACR > 30 mg/g), hypertension (e.g., elevated blood pressure, Stage 1 hypertension, and Stage 2 hypertension), sickle cell trait and disease, hepatitis B & C infection, and Cytomegalovirus (CMV) infection, all stratified by HIV status.
2. Prevalence of *APOL1* variants (high-risk (2 copies of *APOL1* risk variants) and low-risk (0–1 copies of *APOL1* risk variants), by HIV status.
3. Proportion of ART-treated adults with suspected tubular albuminuria (urine albumin-to-protein ratio (uAPR) < 40%) [24, 25] as identified during Aim 1 cross-sectional evaluation.
4. Proportion of ART-treated adults with suspected glomerular albuminuria (albumin-to-protein ratio (uAPR) ≥ 40%) [24, 25] as identified during Aim 1 cross-sectional evaluation

### Laboratory investigations

#### Albuminuria and proteinuria measurement

Urine biomarker assays (e.g., urine albumin, creatinine, and protein measurements) will be performed at the Bonventre laboratory (Brigham and Women’s Hospital Biomarker Laboratory, Boston, MA, USA) using the Randox Imola platform (Crumlin, UK) for urine creatinine measurement and the Randox Imola or BNII nephelometer platforms (Siemens, Malvern, PA) for urine albumin measurement (Bonventre laboratory). Specific urinary measures will be performed using the kinetic Jaffe method (creatinine), pyrogallol red colorimetry method (protein) and immunoturbidimetry (albumin) [78, 79]. Each participant will also provide a serum sample for creatinine, from which we will calculate eGFR using the 2021 CKD-EPI equation, specifically, the 2021 eGFR creatinine (Age and Sex (AS)) equation) [80, 81]. For quality control and assurance (QC/QA) purposes, we will utilize existing laboratory specimen handling and analysis standard operating procedures that we have developed and implemented in collaboration with the Bonventre laboratory team.

Each screened participant will be given a unique study ID number. The study subjects’ IDs can be linked back to participants and will be maintained in REDCap on study-specific secure (encrypted) tablets.

#### Genetics specimen collection and genotyping

Genotyping data are already available for participants who completed the screening phase of the R3 study. Dried blood spot (DBS) samples will be obtained for all newly enrolled participants. DBS samples for genotyping will be obtained by fingerprick, spotted (up to 125μL per each 1 inch application) on Whatman FTA cards (Whatman, Brentford, UK) using capillary tubes and subsequently dried at room temperature for 1 h. We will store the spotted Whatman FTA cards at room temperature (20–25° C) with a desiccant for humidity-control, and these cards will be shipped in batches to the Duke Molecular Physiology Institute (DMPI) Substrate Services Core Facility. Once at DMPI, using sterile biopsy punches, the team will take 6 mm punches from each dried-blood spot sample, with the filter card punch to be placed directly into an extraction tube, and the DNA isolated using Qubit DNA extraction kits (Thermo Fisher Scientific, Waltham, MA). Two elutions will be performed to increase the DNA yield, and DNA will be quantified using PicoGreen dsDNA Assays (Thermo Fisher Scientific, Waltham, MA, USA). For genotyping and sample handling, we will check 2.5–5% of duplicate filter papers for genotype match (and possibly run sex markers) for sex match between clinical record and genotypic sex.

*APOL1* genotyping will be performed in the Olabisi laboratory at the DMPI using TaqMan assays (San Diego, CA) targeting the 3 *APOL1* variants (rs73885319 [G1], rs60910145 [G1], and rs 71785313 [G2]) associated with CKD and HIV-associated kidney disease. The *APOL1* risk alleles are defined by G1 haplotypes (rs73885319-G/rs60910145-G and rs73885319-G/rs60910145-T) and the G2 haplotype (rs7175313-deletion). Testing for the chromosome 11 hemoglobin subunit beta (HBB) sickle cell variants HbS (rs334; p. Glu6Val) and HbC (rs33930165, p. Glu6Lys) will be performed using TaqMan assays. Other genetic loci associated with kidney disease or with susceptibility to infections may also be studied on an exploratory basis. We will define the *APOL1* genotype from the number of risk alleles. In recessive models, individuals exhibiting two risk alleles (G1/G1, G1/G2, or G2/G2) will be assigned to the high-risk (HR) group, while individuals carrying no or one risk allele (G0/G0, G0/G1, and G0/G2) will be assigned to the low-risk (LR) group. We will also perform exploratory analyses to examine the number of risk alleles (0, 1, or 2 alleles).

All information linking participants to personal identifiers will be destroyed once study follow-up and data analysis are completed. Individual participant data to be collected and maintained by de-identified study code include age, ethnicity, sex, occupation, weight, BMI, comorbid medical conditions (hypertension, diabetes, opportunistic infections, syphilis, cancer, cardiovascular disease, and other conditions of potential relevance to study outcomes), current ART regimen (for HIV-positive participants), and concomitant medications. Data on prior and current use of TDF, indinavir, and ritonavir-boosted protease inhibitors will be collected from HIV-positive participants and from review of the medical chart.

### Statistical considerations

#### Sample size

We enrolled 2500 PWH during the screening phase of R3. For these calculations, we assume a similar prevalence of albuminuria among the 500 participants newly recruited for this study. Of note, even if none of the 500 new recruits have albuminuria, the overall proportion among HIV-positive participants will be > 33%, and power calculations (i.e., detectable relative risks) are similar. Table 3 shows, for given sample sizes, the proportion of HIV-negative controls with uACR > 30 mg/g that we would be able to detect with 90% and 80% power at the alpha = 0.05 level (i.e., with a two-sided p-value < 0.05). For example, with 750 HIV-negative controls, we will have ~ 90% power to detect a relative risk for albuminuria between HIV-positive and HIV-negative participants of 1.19 (0.400 vs. 0.336, respectively).

**Table 3:** Detectable proportion with microalbuminuria/macroalbuminuria with various sample sizes

Number of HIV-negative participants (N = 3000 HIV-positive participants)	Detectable proportion with micro-/macroalbuminuria among HIV-negative controls (RR = relative risk)	
	90% power	80% power
300	0.306 (RR = 1.31)	0.319 (RR = 1.25)
500	0.325 (RR = 1.23)	0.335 (RR = 1.19)
**750**	**0.336 (RR = 1.19)**	**0.345 (RR = 1.16)**
1000	0.343 (RR = 1.17)	0.350 (RR = 1.14)

Bolded numbers on far left (750) for each of these tables reflect our chosen final sample size for number of HIV-negative participants to be enrolled

In preliminary data from the R3 cohort, only 4% of participants were LTFU between the two uACR measurements. To account for LTFU, we conservatively estimate that we will need to recruit ~ 810 HIV-negative participants and ~ 540 newly enrolled HIV-positive participants (~ 8% LTFU). We plan to do analyses both categorizing uACR (> 30 mg/g) and treating it as a continuous variable. From our preliminary R3 study data, the mean uACR among HIV-positive adults was ~ 67 mg/g (standard deviation 266 mg/g). If our data are similarly skewed in this study, we will transform the data before analyzing. The mean log_10_ transformed uACR among HIV-positive adults was 1.35 log_10_ (mg/g) with SD = 0.554 log_10_(mg/g). Therefore, with n = 750 HIV-negative and 3000 HIV-positive participants, we anticipate having ~ 90% power to detect a difference in mean log_10_-transformed uACR between HIV-positive and HIV-negative adults of 0.132 standard deviations or 0.07 (i.e., 1.35 log_10_(mg/g) in HIV-positive vs. 1.28 log_10_(mg/g) in HIV-negative adults, corresponding to a median uACR of ~ 22.4 vs. 19.0 mg/g.

The calculations in Table 3 (and Tables 4 and 5) do not account for loss-to-follow-up (LTFU) between the two uACR measurements in the cross-sectional study. In our preliminary data, only 4% of patients were LTFU between the two measurements. Power is greater (although clinical interpretation may be more challenging) when uACR is treated as a continuous outcome. We plan to do analyses both categorizing uACR (> 30 mg/g) and treating it as a continuous variable. We therefore also present power evaluating uACR as a continuous outcome (Table 4). With 750 HIV-negative and 3000 HIV-positive participants, we will have ~ 90% power to detect a difference of 0.132 standard deviations between the mean outcome in HIV-negative vs. HIV-positive adults. From our preliminary data, the mean uACR among HIV-positive adults was ~ 67 mg/g (standard deviation 266 mg/g). This is quite skewed, so we will transform the data before analyzing. The mean log_10_ transformed uACR among HIV-positive adults was 1.35 log_10_ (mg/g) with SD = 0.554 log_10_ (mg/g). Therefore, with n = 750 HIV-negative and 3000 HIV-positive participants, we anticipate having ~ 90% power to detect a difference in mean log_10_-transformed uACR between HIV-positive and HIV-negative adults of 0.07 (i.e., 1.35 log_10_(mg/g) in HIV-positive vs. 1.28 log_10_(mg/g) in HIV-negative adults, corresponding to median uACR of ~ 22.4 vs. 19.0 mg/g.

**Table 4:** Detectable mean difference for continuous outcome (in SDs) between HIV + and HIV- participants

Number of HIV-negative participants (n = 3000 HIV-positive participants)	Detectable difference between mean uACR in HIV-positive and HIV-negative participants (standard deviations)	
	90% power	80% power
300	0.196	0.170
500	0.157	0.135
**750**	**0.132**	**0.114**
1000	0.118	0.102

Bolded numbers on far left (750) for each of these tables reflect our chosen final sample size for number of HIV-negative participants to be enrolled

**Table 5:** Interaction effects based on hypertension

Number of HIV-negative participants	Albuminuria prevalence for HIV-negative participants without hypertension	Albuminuria prevalence for HIV-negative participants with hypertension	Relative Risk (HIV-negative participants)
750	0.10	0.27	2.7
750	0.15	0.36	2.4
750	0.20	0.44	2.2
**750**	**0.25**	**0.50**	**2.0**

#### Power calculations for interaction effects

We will examine whether the association between risk factors and albuminuria differs between HIV-positive and HIV-negative participants. Table 5 illustrates the power for detecting interaction effects based on hypertension. From preliminary R3 data, 12.7% of our study participants had Stage 1 or 2 hypertension (JNC-7 classification). Overall, 47% of HIV-positive adults with hypertension had albuminuria, and 39% of HIV-positive adults without hypertension had albuminuria, for a relative risk of 1.22. If the prevalence of hypertension is ~ 10% among our HIV-negative participants, then Table 5 shows the relative risks among HIV-negative individuals for which we anticipate having ~ 80% power to detect based on varying prevalence of albuminuria. For example, with 750 HIV-negative adults and a true prevalence of albuminuria of 0.25 among HIV-negative adults who do not have hypertension, we have 80% power to detect a difference of relative risks for hypertension of 1.22 vs. 2.0 between HIV-positive and HIV-negative participants, respectively (Bold emphasis row, Table 5).

#### Statistical analysis plan

We will fit regression models based on categorized (i.e., logistic regression) and continuous (i.e., linear regression after log_10_ transformation and/or semiparametric cumulative probability models that empirically estimate the transformation) uACR levels [82]. HIV status is the primary exposure variable. We will perform additional analyses to investigate the interaction between HIV status and individual risk factors, including hypertension and co-infection (HBV and CMV), to determine whether associations differ based on HIV status (e.g., whether the impact of comorbidities on microalbuminuria is greater among PWH). Regression models will be adjusted for potential confounders, including age, ethnicity, smoking status, and BMI. Primary analyses will control for sex and will be repeated among men and women, separately, to investigate potential differences by sex. For the analyses limited to PWH, viral load (detectable/undetectable) will be included in models as a potential confounder. Missing data will be multiply imputed using standard techniques. Continuous covariates will be expanded using restricted cubic splines with three knots to avoid linearity assumptions. Additional analyses will be performed in the subset of HIV-positive adults to assess the association between HIV-specific factors (e.g., TDF exposure, duration on ART, detectable viral load, and CD4 cell count) on albuminuria/uACR (Additional file 1 and Additional file 2).

#### Genetic analysis plans

We will determine the prevalence of *APOL1* risk alleles, comparing the 750 HIV-negative adults to our 3000 HIV-positive study participants, specifically comparing the proportions of each population (i.e., HIV-negative vs. HIV-positive) possessing 0, 1, and 2 *APOL1* risk haplotypes (G1 or G2). We will examine the association between *APOL1* genotype and uACR and eGFR, adjusting for sex, age, and HIV status. Furthermore, we will test whether the associations between *APOL1* genotype and our outcomes differ by HIV status by including interaction terms. Based on prior genetic association work in HIV-associated kidney disorders [83–89], we will test both additive and recessive genetic models.

### Specific aim 2

#### Study population

The *inclusion criteria* are as follows:

- Participants with normoalbuminuria (uACR < 30 mg/g) who complete Aim 1 are eligible for Aim 2 (Fig. 1).
- Participants that completed Aim 1 of the separate, ongoing R3 study [13, 14] having normoalbuminuria (uACR < 30 mg/g) based on the average of two previously obtained uACR measurements (4–8 weeks apart) and re-confirmed via an additional uACR measurement obtained at time of screening for this study are also eligible.

#### Exclusion criteria

- Did not complete all scheduled Aim 1 visits
- Pregnancy (all women with reproductive potential will undergo urine pregnancy testing)
- Diabetes mellitus (documented diagnosis or random glucose ≥ 6.1 mmol/L [110 mg/dL])
- Known congenital kidney disease and/or autoimmune disease that can cause albuminuria
- Actively taking ACEi or ARBs, which may suppress albuminuria
- Regular use of NSAIDs or traditional medications
- HIV-negative but use TDF as pre-exposure prophylaxis or HBV treatment
- Comorbid condition with life expectancy < 3 years
- Planning to relocate within three years

#### Study procedures

Whole blood and urine biospecimens will be obtained for processing for plasma and urine pellets which will be processed and shipped to their respective research laboratories, namely, Duke University (plasma biomarker, *Cytomegalovirus* (CMV) IgG antibody (Ab) testing, and *APOL1* genotyping) and Brigham and Women’s Hospital (urine biomarkers and urine albumin, creatinine, and protein testing). Participants will receive reimbursement for comprehensive study visits. All HIV-negative study participants will be asked to complete a brief risk assessment at each study visit, with reflex viral load testing in those deemed high risk, as outlined in Aim 1 (study procedures). If their HIV test results are positive, they will no longer participate in longitudinal follow-up. Data collected prior to seroconversion will be maintained. Longitudinal assessments (enrollment visit and then every 6 months for 30 months for a total of 5 scheduled visits for each participant) will include the following (*as outlined below*):

#### Study measures

*Demographic and clinical data elements* will be abstracted from medical record and by self-report at baseline and will be updated during every scheduled study visit.

- *Demographics*: age, sex, self-reported ethnicity (Hausa/Fulani, Igbo, Yoruba, Other (Specify)
- *Education*: not literate, primary, secondary, tertiary (university or other college).
- *Occupation*: employment status (and if employed; details to be obtained)
- *HIV transmission risk factors*: heterosexual activity, homosexual activity, transfusion, other, unknown
- *Date of HIV diagnosis (PWH only)*: date of first positive HIV test
- *Date of ART initiation (PWH only)*: defined as three or more active antiretrovirals from two or more drug classes
- *Dates of AIDS-defining illnesses (PWH only)*: defined per CDC list of AIDS-defining illnesses
- *Dates of comorbid disease diagnoses*: infections including TB (pulmonary and extrapulmonary), viral infections (HBV, HCV [Aim 1], Lassa fever, COVID-19, etc.), AIDS-defining illnesses; NCDs including hypertension, diabetes, sickle cell trait or disease, dyslipidemia, cardiovascular disease, cancer, liver disease, lung disease, depression, and bone mineral density abnormalities (including non-traumatic fractures).
- *Antiretroviral medication start/stop dates (PWH only)*: from clinical record and pharmacy data (evaluating current exposure as well as prior/current TDF, protease inhibitor use to determine cumulative exposure time).
- *Current medications*: all medications taken at least once daily for ≥ 30 days, including traditional medications.
- *Laboratory HIV data*: all CD4 cell count and viral load measurements (collected per standard of care)**.** SARS-CoV-2 testing will be performed per existing standard of care and results of all COVID-19 screening tests will be captured in the patients’ study medical record).

*Social and behavioral data* will be collected three times during study follow-up using the following scales, which are all available in both English and Hausa languages (Table 6).

- *Substance use:* The Alcohol, Smoking and Substance Involvement Screening Test (ASSIST) is a cross-cultural screening of recent and lifetime substance use [90].
- *Depression:* The 9-item Patient Health Questionnaire (PHQ-9) [91] screens for depressive symptoms.

**Table 6:** Procedures for longitudinal cohort of HIV-positive (n = 1,000) and HIV-negative (n = 500) participants with normoalbuminuria to be followed for 30 months (n = 1500 total)

Procedure / Visit (month (mo))	Enrollment	6 mo	12 mo	24 mo	30 mo
Seated blood pressure check	X	X	X	X	X
Diabetes mellitus screening (perform fasting blood glucose if any signs/symptoms suggestive of underlying diabetes)	X^*^	X	X	X	X
uACR^a^ (No urine protein testing necessary for enrolled Aim 2 HIV-positive adults with normoalbuminuria as all HIV-positive ART-treated adults from Aim 1 will have one uAPR measurement) (We will have stored urine at 3 timepoints to go back and perform uAPR testing in patients with incident microalbuminuria)	X^#^ (We will use Aim 1 uACR as participants baseline value)		X		X
Serum creatinine (SCr)^b^	X (We will use Aim 1 SCr as their baseline value)		X		X
Parasite screening					
Complete blood count (CBC) with differential^c^	X	X	X	X	X
Urine dipstick (spun for all patients with hematuria followed by urine microscopy (*S. haematobium*)	X	X	X	X	X
Malaria testing (rapid diagnostic test) at point-of-care (POC) using fingerprick specimen	X	X	X	X	X
Filariasis POC testing for *Wuchereria bancrofti*^d^	X				X
Onchocerciasis testing (bilateral skin snips)^e^	X				X
Stool for ova and parasite (stool O&P)^f^	X	X	X	X	X
Daytime thick blood smear for *Loa loa* (10 AM–2 PM)	X				X
Strongyloides IgG serology (plasma)^g^	X				X
Tuberculosis screening^h^	X	X	X	X	X
Inflammatory biomarkers (urine and plasma)^i^	X				X
CD4 cell count	X				X
Viral load (plasma HIV RNA)	X				X
Social and Behavioral data	X		X		X
Additional tubes for storage^j^	X		X		X

*All potentially eligible Aim 2 participants will undergo fasting blood glucose testing to evaluate for diabetes (and if ≥ 6.1

mmol/L (110 mg/dL), then patient may have diabetes, is ineligible, and is referred to medical provider/clinic for evaluation

^#^All participants will use their recent uACR measurement (obtained in Aim 1) for their baseline

^a^Two 3 mL sterile universal containers taken for uACR testing (Urine protein testing only for HIV-positives)

^b^Two 3 mL gold top tubes taken to measure serum creatinine (SCr)

^c^CBC with differential—looking for eosinophilia (common with Filariasis (especially *Loa loa*) and Schistosomiasis)

^d^POC testing for *W. bancrofti* antigen by Filariasis Test Strip (FTS), followed by obtaining nocturnal thick blood smears for

microfilaria [between 10 pm—2 am] in FTS-positive persons. *Brugia spp. *are not endemic to Africa, so testing for *W*

*bancrofti* will be sufficient

^e^*Onchocerca volvulus *ELISA is not sensitive enough to rule out infection. Physical exam findings are also not sensitive. No one

will want to be snipped every 6 months, but since filarial worms are long-lived (> 5 years), snipping at the beginning and end

of study should capture anyone who is infected at study onset or becomes infected during the study

^f^Stool for O&P to be done screening for *S. mansoni* and soil transmitted helminths (e.g., Ascaris, Trichuris, Hookworm)

^g^Strongyloides IgG ELISA at baseline in ALL participants & test all persons testing NEG at enrolment again at 30 months

^h^Sputum specimens (5 mL) to be collected and sent for Gene Xpert testing and/or microscopy (acid fast bacilli staining)

^i^Two 3 mL purple top tubes for plasma biomarkers and one 5–7 mL urine specimens for urine biomarkers (and stored)

^j^Two 3 mL purple top EDTA tubes and one 5–7 mL urine specimen to be obtained and stored for future/approved testing

*Clinical Assessments* at all comprehensive study visits will be performed by trained study nurses and will include:

- *Vital signs:* temperature, respiratory rate, heart rate, blood pressure (Seated BP checked every study visit using a validated Omron series 5 upper arm digital BP device).
- *Anthropometrics*: height, weight, waist circumference
- *Urine albumin testing*, *uACR measurements*: Albuminuria will be assessed at each visit and categorized as defined in Aim 1. Subjects with incident macroalbuminuria will be referred to a clinician for evaluation and to a nephrologist for additional testing/management, per standard of care.
- *Urine albumin-to-protein [uAPR] ratios (HIV-positive participants)*: will be assessed to screen for possible TDF-mediated tubular insult as a contributing cause of microalbuminuria. Subjects with *uAPR* < *40%* will be classified as *tubular albuminuria* and those with *uAPR* ≥ *40%* will be classified as *glomerular albuminuria*.
- *Diabetes screening*: All potentially eligible Aim 2 participants will undergo fasting/random blood glucose testing to evaluate for diabetes (and if ≥ 6.1 mmol/L (110 mg/dL), then patient may have diabetes, is ineligible, and is referred to medical provider/clinic for evaluation. Once enrolled, during each study visit, participants will be screened for symptoms of diabetes. In those with symptoms, we will perform fasting/random blood glucose testing. if ≥ 6.1 mmol/L (110 mg/dL), they will be immediately referred to a specialist for further evaluation.

#### Co-infection screening

- *Mycobacterium tuberculosis* (TB): Sputum specimens for acid fast bacilli (AFB) microscopy and/or Gene Xpert testing in patients having a cough of ≥ 2 weeks in duration, weight loss, and/or night sweats). Mycobacterial blood cultures, lymph node aspirates, body fluid (i.e., thoracentesis) and imaging studies will be obtained per standard of care when evaluating for suspected extrapulmonary TB.
- *Parasitic infections* (see Table 6) (The results obtained from enrollment filariasis (*W. bancrofti, O. volvulus, and L. loa*) screening will determine the need for repeat testing at the 30-month visit (i.e., if prevalence is extremely low, we will not re-test at the 30-month visit).

#### *Adverse clinical outcomes* will be assessed at all study visits by trained study research staff

- *Hospitalizations:* We will ask all participants if they have been hospitalized in the previous 6 months. If they answer affirmatively, we will ask the reason for hospitalization and approximate dates.
- *Death*: Ascertained from medical records or from a contact identified by the participant during consent.

- *Inflammation* will be assessed from plasma and urine specimens collected, as detailed in Table 6. Plasma inflammatory biomarker assay testing will be performed in duplicate at the DMPI Biomarker Core Facility using the Mesoscale Discovery Platform. Based on the literature, we chose a panel of plasma and urine biomarkers [92–101] that will help us elucidate the processed by which chronic inflammation contributes to albuminuria. Urine inflammatory/tubular injury biomarker assay testing will be performed at the Bonventre laboratory (Brigham and Women’s Hospital Biomarker Laboratory, Boston, MA, USA). Some are novel biomarkers whose role in clinical research is being defined.
- *Plasma inflammatory markers:* highly sensitive C-reactive protein (hsCRP), IL-6, soluble TNF-receptor 1 (TNFR-1), soluble TNF-receptor 2 (TNFR-2), IL-1β, and TNF (with biomarker levels of sCD14, fibrinogen, and possibly additional plasma biomarker levels to be ascertained pending additional funding/approved sub-studies).
- *Urine inflammatory/tubular injury biomarkers*: NGAL, KIM-1, and MCP-1 (with biomarker levels of IL-18, interferon-gamma inducible protein-10 (IP-10), and possibly additional urine biomarker levels to be ascertained pending additional funding/approved sub-studies).

#### Study outcomes

1. Proportion of Aim 2 longitudinal cohort participants experiencing progression from normoalbuminuria (uACR < 30 mg/g) to microalbuminuria (uACR = 30–300 mg/g), at 30 months of follow-up, overall, and by TB infection, parasitic infection, and inflammation (high levels of plasma and/or urine inflammatory marker levels) status.
2. Proportion experiencing a doubling of serum creatinine.
3. Proportion experiencing a decline in eGFR > 30% at 30 months (using the 2021 CKD-EPI eGFR creatinine (age and sex)) equation.
4. Mean change in eGFR at 30 months (using the 2021 CKD-EPI eGFR creatinine (age and sex)) equation.
5. Mean change in uACR at 30 months.

### Statistical considerations

#### Sample size

We will enroll 1500 (1000 HIV-positive and 500 HIV-negative) patients with normoalbuminuria and follow them for up to 30 months. Table 7 summarizes differences in albuminuria incidence that we will have 80% power to detect between the HIV-positive and HIV-negative groups, over a range of sample sizes and albuminuria incidence rates in the HIV-positive group. We will have approximately 80% power to detect a difference of developing albuminuria of 10% (HIV-positive) vs. 5.9% (HIV-negative), or a relative risk of about 1.70 (Bold emphasis row in Table 7).

**Table 7:** Detectable alternative for various rates of developing albuminuria for the HIV-positive adult patients

HIV-positive adults, N	HIV-negative adults, N	3-year incidence of albuminuria for HIV-positive adults	3-year incidence of albuminuria, HIV-negative adults, 80% power to detect	Relative Risk
1000	500	5%	2.2%	2.28
**1000**	**500**	**10%**	**5.9%**	**1.70**
1000	500	15%	10.0%	1.51

#### Statistical analysis plan

Demographic and clinical characteristics will be summarized by descriptive statistics. We will perform two sets of analyses: (1) categorizing uACR and investigating time from enrollment to albuminuria onset (first uACR measurement > 30 mg/g), and (2) keeping uACR as a continuous variable and investigating mean change in repeated uACR measures. Categorization of uACR is common in the literature and allows easy clinical interpretation, whereas continuous analysis tends to result in greater power. For (1), discrete-time proportional hazards models with a complementary log–log link will be fit. For (2), generalized estimating equations (GEE) with an identity link (i.e., linear model) will be fit to repeated measures of log_10_-transformed uACR. We will fit models limited to PWH, with cumulative TDF exposure as the primary (time-varying) exposure. The association between albuminuria and cumulative exposure to other regimens (e.g., protease inhibitors) will also be considered, both in models with and without cumulative TDF exposure. Analyses will adjust for viral suppression status. Secondary analyses will exclude the anticipated small proportion (< 5%) that will not be virally suppressed.

For hypothesis (ii), we will fit models with HIV status, hypertension, and an interaction between HIV status and hypertension. For hypothesis (iii), we will fit models with time-updated biomarkers of inflammation, both current and cumulative exposure. For inflammatory analyses, we will compute a composite inflammation score [102] for each participant that has been shown to accurately predict the phenotype of interest, based on levels (at least initially) of select markers, with a score of 1 being assigned to each individual biomarker measurement deemed “*high*” (or “*significantly elevated*”) delineated as follows: hsCRP > 3 mg/L, IL-6 > 6 pg/mL, TNF ≥ 7 pg/mL, and IL-1β ≥ 0.39 pg/mL. We will also perform analyses including inflammation biomarkers as continuous variables, both individually and in combination with other biomarkers.

Urine and plasma biomarkers will be collected and stored on all Aim 2 study participants, but we will perform case–control analyses using cases defined as participants who develop incident albuminuria during follow-up (i.e., incident cases) and compare their biomarker profiles to a matched set of non-albuminuric controls. We conservatively estimate that we will observe ~ 130 incident cases; we plan to match three controls to each case based on age (± 10 years), sex, HIV-status, CD4 cell count at enrollment among those HIV-positive (within 50 cells/mm^3^), and hypertension status. If matching on this many factors proves to be difficult, then we will consider matching on the predicted probability of being a case based on the factors listed above (i.e., propensity score-type matching). We will account for the case-cohort nature of the design with appropriate analyses that account for outcome-dependent sampling [103].

All analyses will account for confounding variables, including time-updated viral suppression status (only participants with suppressed viral load will be enrolled), smoking status, BMI, CD4 cell count, age, exposure to TDF or protease inhibitors, etc. We anticipate using propensity score matching weights [104], to account for the high number of confounders, particularly in our time-to-albuminuria analyses (which will likely not have enough events to allow inclusion of all potential confounders). In all analyses, to relax linearity assumptions, continuous predictors will be expanded using restricted cubic splines.

Missing data, which we assume will be minimal, will be imputed using multiple imputation procedures. The longitudinal nature of our data collection may also permit investigation of mediation (e.g., hypertension → inflammation → albuminuria), which will be explored using relevant methods [105].

## Discussion

*APOL1* high risk genotype and traditional risk factors for kidney disease (e.g., hypertension, diabetes, smoking) do not fully explain the high rates of albuminuria seen in our HIV-positive, ART-experienced adult Nigerian population, although hypertension is likely to be an important contributing factor. Other factors that may contribute to albuminuria can be broken down into three main categories, two of which will be evaluated in-depth in this study: i) *direct kidney injury* (i.e. exposure to the ARV medication TDF, which can cause tubulopathy); ii) *immune activation/inflammation* from HIV or specific viral pathogens (CMV, HBV, or HCV) and/or other endemic co-infections (i.e. parasitic infestations, tuberculosis); and iii) *environmental factors/exposures* (i.e. traditional medications, heavy metals, etc.).

*This study is innovative for the following reasons*: (i) The findings of this study, depending on which factors are identified as contributing most to microalbuminuria, will guide the design of interventions aimed at identifying and more aggressively managing microalbuminuria in PWH earlier in their risk trajectory towards overt cardiovascular and kidney disease; (ii) This will be the first study in sub-Saharan Africa to comprehensively evaluate the interplay between genetic susceptibility (*APOL1* risk alleles), comorbid traditional risk factors, and chronic inflammation/immune activation and endemic co-infections on microalbuminuria. Prior studies in this population have rarely considered the role of comorbid sickle cell trait/sickle cell disease or the impact of co-infections other than HBV or HCV.

The anticipated findings from this study will support the development of interventions to address microalbuminuria in PWH to reduce kidney and cardiovascular morbidity and mortality, including more intensive monitoring and treatment of traditional risk factors, provision of renin-angiotensin aldosterone system or sodium-glucose cotransporter-2 inhibitors, consideration of changes in ART regimen, and screening and treatment for relevant co-infections.

## Trial status

The current version of the protocol is version 6.0., with enrollment slated to commence in July 2022. We anticipate enrollment for aim 1 to be completed by October 1, 2022, and enrollment for aim 2 to be completed by December 31, 2022.

## Supplementary Information

**Additional file 1. **SPIRIT Checklist for Trials.

**Additional file 2.** SPIRIT Figure.

## Data Availability

Deidentified patient- and study-level data as well as the study protocol, underlying the study findings from this study (when available), will be shared by the corresponding author on request. Analysis scripts will be posted at https://biostat.app.vumc.org/wiki/Main/ArchivedAnalyses. De-identified and linked phenotype and genotype data generated from consenting human subjects in this study will be posted on a publicly accessible repository, such as dbGaP, with appropriate access controls.

## Abbreviations

AKTH: Aminu Kano Teaching Hospital; Kano, Nigeria-study site
*APOL1* HR: Apolipoprotein-1 high-risk genotype
*APOL1*: Apolipoprotein-1
ARB: Angiotensin receptor blocker
ART: Combination antiretroviral therapy
ASSIST: Alcohol, Smoking and Substance Involvement Screening Test screening tool
ARV: Antiretroviral medication.
BMI: Body mass index.
CD4: CD4 helper cell/CD4 cell count
CKD: Chronic kidney disease
CKDEPI: Chronic kidney disease epidemiology collaboration
CMV: Cytomegalovirus
CrCl: Calculated creatinine clearance
CyC: Serum cystatin C
EDTA: Ethylenediaminetetraacetic acid (purple top tube)
eGFR: Estimated glomerular filtration rate
ELISA: Enzyme-linked immunosorbent assay
ESKD: End-stage kidney disease
FSGS: Focal segmental glomerulosclerosis
GEE: Generalized estimating equation
HBV: Hepatitis B virus
HCV: Hepatitis C virus
HIV: Human immunodeficiency virus
HR: High-risk (*APOL1*) genotype
hsCRP: Highly sensitive C-reactive protein
IL-1β: Interleukin-1 Beta
IL-6: Interleukin-6
JNC: Joint national committee (for blood pressure classification)
LR: Low-risk (*APOL1*) genotype
LTFU: Lost to follow-up
PHQ-9: Patient health questionnaire; depression screening tool
PLHIV: People living with HIV
POC: Point-of-care
R3: Renal Risk Reduction study
RAAS: Renin-angiotensin aldosterone system
RCT: Randomized controlled trial
SCr: Serum creatinine
SD: Standard deviation(s)
TAF: Tenofovir alafenamide
TDF: Tenofovir disoproxil fumarate
TLD: Tenofovir (TDF), lamuvidine (3TC), plus dolutegravir (DTG)
TNF: Tumor necrosis factor
uACR: Urine albumin-to-creatinine ratio
uAPR: Urine albumin-to-protein ratio
VL: Viral load (HIV-1 RNA level)
