# Supplementary material for: Etiology of Persistent Microalbuminuria in Nigeria (P_MICRO study): protocol and study design
Source: BMC Infect Dis. 2022 Jul 4;22:591. doi: 10.1186/s12879-022-07531-y (PMC9251938; doi:10.1186/s12879-022-07531-y)
Supplement: Supplementary file 2 — Additional file 2. SPIRIT Figure. [file 12879_2022_7531_MOESM2_ESM.doc]

**SPIRIT Figure**

|  | **STUDY PERIOD** | | | | | | |
| --- | --- | --- | --- | --- | --- | --- | --- |
|  | **Before start of follow-up** | **Baseline** | **Follow-up over 24 months** | | | |  |
| **TIMEPOINT** | ***-t_1_*** | **0** |  | | | | ***t_1_*** |
| **ENROLMENT:**  **Ethics Approvals** | X |  |  |  |  |  | |
| **Hire and Train Study Staff** | X |  |  |  |  |  | |
| **Informed consent** | X |  |  |  |  |  | |
| ***Screening and Enrollment for Aim 1*** | X |  |  |  |  |  | |
| ***Enrolment for Aim 2*** | X |  |  |  |  |  | |
| **Data Collection** |  | X | X | X | X | X | |
| **INTERVENTIONS:** |  |  |  |  |  |  | |
| ***Follow-up*** |  |  |  |  |  |  | |
| **ASSESSMENTS:** |  |  |  |  |  |  | |
| ***Urine Albumin-Creatinine Ratio***  ***Glomerular Filtration Rate*** |  | X |  | X |  | X | |
| ***Social/Behavioral Data*** |  | X |  | X |  | X | |
| ***Parasite Screening*** |  | X | X | X | X | X | |
| ***Plasma and Urine Inflammatory Biomarkers***  ***CD4 cell count / Plasma HIV-1 RNA (Viral Load)*** |  | X |  |  |  | X | |
| **Data Analysis** |  |  |  |  |  | X | |
| **Dissemination of study findings** |  |  |  |  |  | X | |
